# Topical Skin Application of Small-Molecule Antiplatelet Agent against Pressure Injury in Rat Models

**DOI:** 10.3390/ijms25031639

**Published:** 2024-01-29

**Authors:** Yuan Yuan, En Takashi, Ping Hou, Akio Kamijo, Daiji Miura, Hirotomo Ten

**Affiliations:** 1 Division of Basic & Clinical Medicine, Faculty of Nursing, Nagano College of Nursing, Komagane 399-4117, Nagano, Japan; 007257@yzu.edu.cn (P.H.); a.kamijo@nagano-nurs.ac.jp (A.K.); miura-daiji@nagano-nurs.ac.jp (D.M.); 2 School of Nursing and Public Health, Yangzhou University, Yangzhou 225000, China; 3 Department of Judo Physical Therapy, Faculty of Health, Teikyo Heisei University, Tokyo 170-8445, Japan; ten@nms.ac.jp

**Keywords:** pressure injury, antiplatelet agent, against injury, topical application, small molecule

## Abstract

Due to prolonged forced positioning, the incidence of intraoperative pressure injuries is high. This study aimed to explore the impact of small-molecule antiplatelet drugs on pressure injuries by locally applying them before an injury occurs. In the first part of this study, water-soluble tracers with different molecular weights were applied to normal and early-stage pressure-injured skin. Through digital cameras, spectrophotometers, and histological observations, the penetration of tracers into the epidermis was clarified. In the second part of this study, a water-soluble antiplatelet drug called Trapidil (molecular weight = 205 Da) was applied to the left side of the back of a rat before, during, and after compression, and the contralateral side served as a non-intervention control group. The differences in pressure injuries between the two groups were observed through a digital camera, an ultraviolet camera, and temperature measurement, and skin circulation and perfusion were assessed via an intravenous injection of Evans Blue. The first part of this study found that water-soluble tracers did not easily penetrate normal skin but could more easily penetrate pressure-damaged skin. The smaller the molecular weight of the tracer, the easier it penetrated the skin. Therefore, in the next step of research, water-soluble drugs with smaller molecular weights should be selected. The second part of this study found that, compared with the control group, the occurrence rates and areas of ulcers were lower, the gray value was higher, and the skin temperature was lower in the Trapidil group (*p* < 0.05). After the intravenous Evans Blue injection, skin circulation and perfusion in the Trapidil group were found to be better. In conclusion, this study found that the topical skin application of a small-molecule antiplatelet agent may have significant effects against pressure injuries by improving post-decompression ischemia, providing new insights into the prevention and treatment of intraoperative pressure injuries.

## 1. Introduction

Pressure injuries (PIs) are defined as local injuries of the skin and/or underlying tissues caused by pressure or pressure combined with shear forces [1]. Pressure injuries not only prolong the recovery time and hospitalization time related to the patient’s primary diseases but also aggravate the condition and increase the difficulty of treatment and the risk of infection. If the infection spreads, it may lead to a serious threat to life [2]. At present, the commonly used prevention methods against PIs, such as timed body position changes and decompression measures, are all means to reduce pressure ischemia [3]. These methods are very effective and significantly reduce the occurrence of PIs [4]. However, for patients in the perioperative period, the above methods are difficult to implement due to the requirements of the surgical position and the surgical field of vision and the fact that many patients are unconscious after the use of anesthetics [5]. Therefore, the operating room is a high-incidence unit of PIs. According to previous research reports, the incidence of acquired PIs in the operating room varies from 8.5% to 66% [6,7]. Therefore, the prevention of perioperative pressure injuries is an urgent and challenging clinical issue.

In recent years, decompressed ischemia has also been considered to be the main cause of pressure injuries. Parish et al. [8] pointed out that the deformation of skin tissue caused by pressure or shear force resulted in excessive stretching and injury in vascular endothelial cells. Recently, Chen et al. [9] put forward the concept of post-decompression ischemia in early PIs, pointing out that this is not the continuation of pressure ischemia but an important pathological state caused by the mechanisms of thrombosis and independent of other circulatory disorders. Other studies have found that thrombosis is not found in rapidly cured pressure injuries. Conversely, thrombosis is reported in delayed pressure injuries. The circulatory disturbance caused by thrombosis has a great impact on the occurrence and deterioration of pressure injuries [10]. It has also been reported that intravenous infusion or intramuscular injection of adrenocorticotropic hormones may reduce the occurrence of pressure injuries and prevent deterioration by inhibiting thrombosis [11]. This suggests that the prevention of pressure injuries may be achieved by inhibiting thrombosis to maintain sufficient blood flow after decompression. The use of antiplatelet drugs is also an effective method for inhibiting thrombosis [12]. 

The systemic use of antiplatelet drugs is generally prohibited during the perioperative period. In order to avoid affecting the blood coagulation function of the whole body and effectively make the drugs reach the PI skin [13], topical skin medication, which is limited to a small area, is an ideal approach. This is because the pressure point during the perioperative period is generally fixed and predictable. However, the main problem in the local administration of drugs on the skin is whether there is enough penetration [14]. Skin is composed of epidermis, dermis, and subcutaneous tissue, which is a protective layer that maintains the stability of the internal environment in an organism. The stratum corneum (SC) at the outermost part of the epidermis is the most important barrier to the percutaneous absorption of drugs because of its special organizational structure. Its existence reduces the permeability and bioavailability of local and percutaneous drug delivery [15]. Studies have shown that drugs with a molecular weight of less than 500 Da and high lipophilicity can more easily pass through the SC [16]. Another study showed that the maximum molecular weight of fat-soluble substances that can penetrate into normal skin is 414 Da, but the maximum molecular weight of the fat-soluble substances that can penetrate into the skin is 986 Da [17] after the SC is damaged by the adhesive tape-peeling test. Another study showed that the skin below the SC shows higher hydrophilicity than the SC [18]. Therefore, we speculate that, for skin with PI, because its skin barrier function is also damaged, it is possible that water-soluble substances with small molecular weights can penetrate through the SC and into the dermis or the skin below the dermis and can effectively play the role of drugs.

Trapidil is an antiplatelet drug with a molecular weight of 205 Da, with extensive biological activity [19], which is generally taken orally. However, intraoperative bleeding caused by the systemic application of antiplatelet drugs is fatal, so oral antiplatelet drugs are often contraindicated before operation [20]. Generally speaking, the compression site during the operation can be predicted before the operation [21]. The local application of dressings and ointments can make the drug penetrate the skin and play a local role near the application site [22], which can prevent the risk of bleeding during an operation. Therefore, we assume that the local application of Trapidil on the compression site before, during, and after the operation not only inhibits thrombosis and reduces the occurrence and deterioration of pressure injuries during the perioperative period but also does not affect the systemic coagulation mechanism. However, the main problem of transdermal drug delivery is whether there is enough penetration. It has been reported that highly lipophilic drugs with a molecular weight of less than 500 Da can more easily enter the SC [16], and SC is the most significant obstacle affecting drug penetration [23]. Although the molecular weight of Trapidil is only 205 Da, it is not known whether its hydrophilic properties affect the transdermal effect.

Therefore, in this study, water-soluble tracers with different molecular weights were first used to observe the transdermal effect, and then Trapidil was topically applied before, during, and after a pressure injury was produced to explore its effect against pressure injuries in rat models.

## 2. Results

### 2.1. Experiments on Penetration of Water-Soluble Tracers with Different Molecular Weights into Skin

#### 2.1.1. Macroscopic Picture of Three Different Tracers Applied on Normal Skin and PI Skin

**Figure 1:**
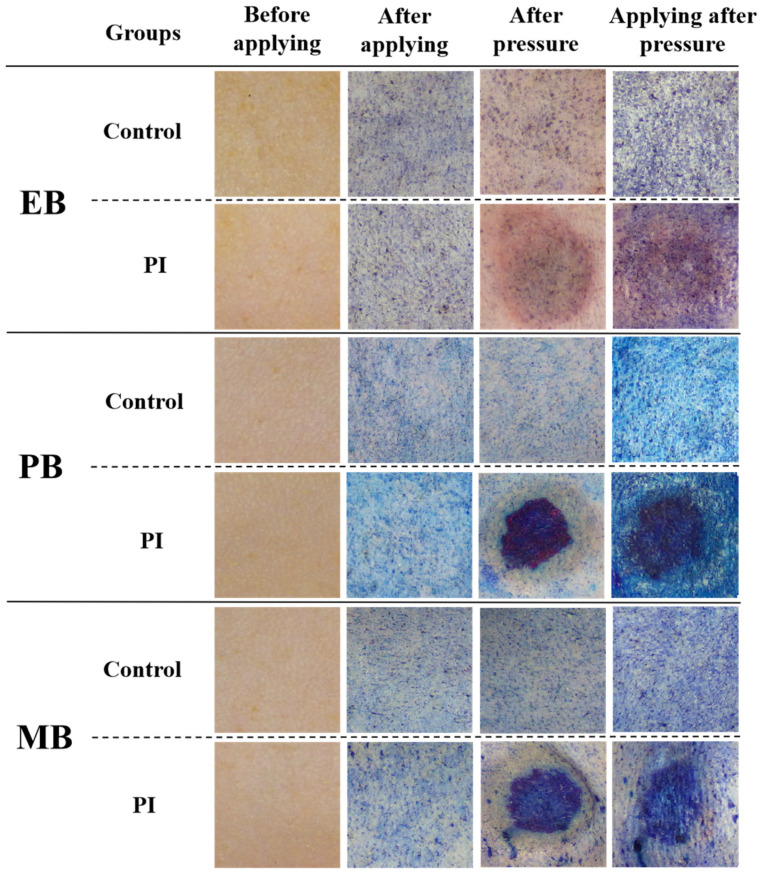
Macroscopic picture of three different tracers applied on normal skin and PI skin. PI: pressure injury. EB: Evans Blue. PB: Patent Blue. MB: Methylene Blue.

As shown in Figure 1, three different tracers, Evans Blue (EB, molecular weight = 961 Da), Patent Blue (PB, molecular weight = 567 Da), and Methylene Blue (MB, molecular weight = 374 Da), were applied on normal skin and PI skin. It can be seen that PB and MB with smaller molecular weights could more easily penetrate the skin, and tracers could more easily penetrate PI skin than normal skin.

#### 2.1.2. Reflectance of Skin Coated with Three Tracers of Different Molecular Weights

**Figure 2:**
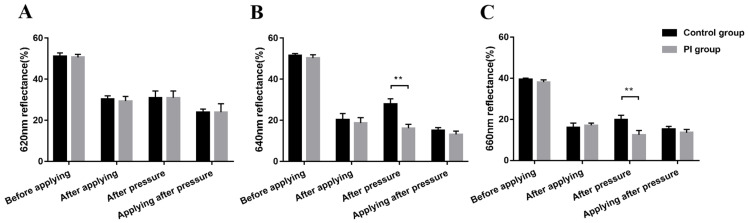
Reflectance of skin coated with three tracers of different molecular weights. PI: pressure injury. ** *p* < 0.01. (**A**) Reflectance of skin coated with EB. (**B**) Reflectance of skin coated with PB. (**C**) Reflectance of skin coated with MB.

As shown in Figure 2, the reflectance of the skin coated with three different tracers was measured. Previous studies have shown that the lower the reflectance, the stronger the penetration. It also proved that PB (Figure 2B) and MB (Figure 2C) with smaller molecular weights could more easily penetrate the skin and that tracers could more easily penetrate PI skin than normal skin.

#### 2.1.3. Histological Observation of Skin Coated with Three Tracers of Different Molecular Weights

**Figure 3:**
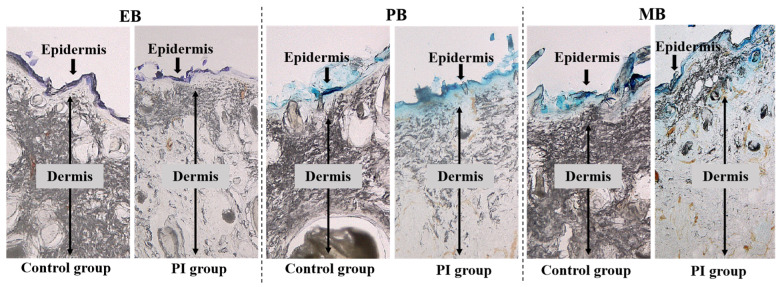
Histological observation of skin coated with three tracers of different molecular weights. PI: pressure injury. EB: Evans Blue. PB: Patent Blue. MB: Methylene Blue.

Figure 3 shows the histological observation of skin coated with three tracers of different molecular weights. In the skin with PI, MB with a molecular weight of 374 Da was the most likely to penetrate the skin, followed by PB with a molecular weight of 567 Da, while EB with a molecular weight of 961 Da hardly penetrated the skin. In the control group, the three tracers basically did not penetrate the normal skin but were confined to the SC of the epidermis.

This part of the study proved that the smaller the molecular weight, the easier it was to penetrate into the skin, and the water-soluble substances could not easily penetrate normal skin but could easily penetrate the skin with PI. Therefore, in the following study, it was better to select water-soluble substances with lower molecular weights. Trapidil, selected as an antiplatelet drug in the second part of the study, is a water-soluble substance with a small molecular weight of 205 Da.

### 2.2. Experiments on Topical Skin Application of Trapidil against PI

#### 2.2.1. Changes in PI Skin

**Figure 4:**
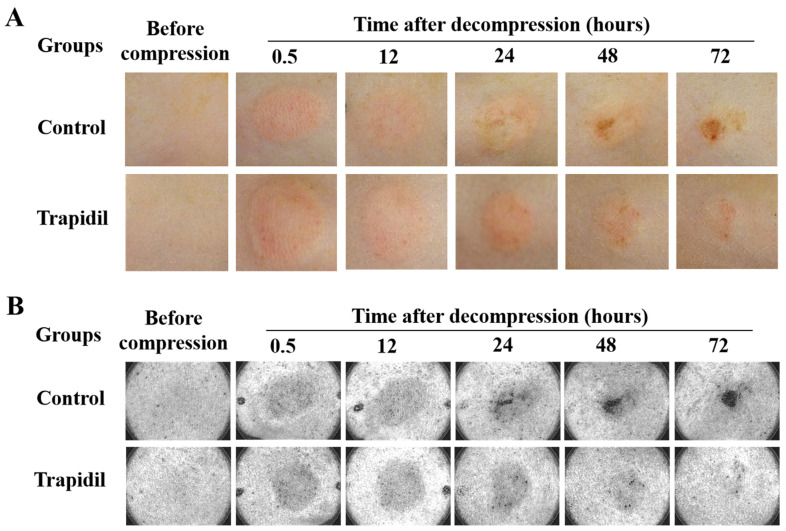
Changes in PI skin in control and Trapidil groups using a digital camera (**A**) and a UV camera (**B**).

The changes in PI skin in the control and Trapidil groups monitored using a digital camera and an ultraviolet (UV) camera are shown in Figure 4. Early PIs were found in both the control and Trapidil groups within 0.5 h after the decompression, and within 48 h, ulcers were noted. The ulcers in the control group were more obvious.

#### 2.2.2. Occurrence Rates and Areas of Ulcers

In terms of the occurrence rates of ulcers, as shown in Table 1, no ulcer was found in either group within 30 min after decompression. At 48 h and 72 h after decompression, the occurrence rate of ulcers in the Trapidil group was significantly lower than that in the control group, and at 48 h after decompression, the difference between the two groups was statistically significant (*p* < 0.05).

**Figure 5:**
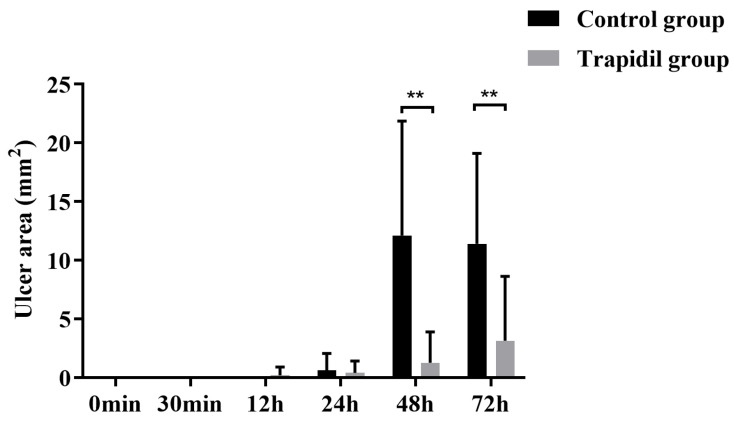
Areas of ulcer in control and Trapidil groups. ** *p* < 0.01.

As shown in Figure 5, areas of ulcers were not found in either group after 30 min of decompression. At 48 h and 72 h after decompression, the areas of ulcers in the Trapidil group were (1.28 ± 2.62) mm^2^ and (3.15 ± 5.48) mm^2^, the areas of ulcers in the control group were (12.12 ± 9.75) mm^2^ and (11.40 ± 7.72) mm^2^, and the differences were statistically significant (all *p* < 0.01).

#### 2.2.3. Severity of PI

**Figure 6:**
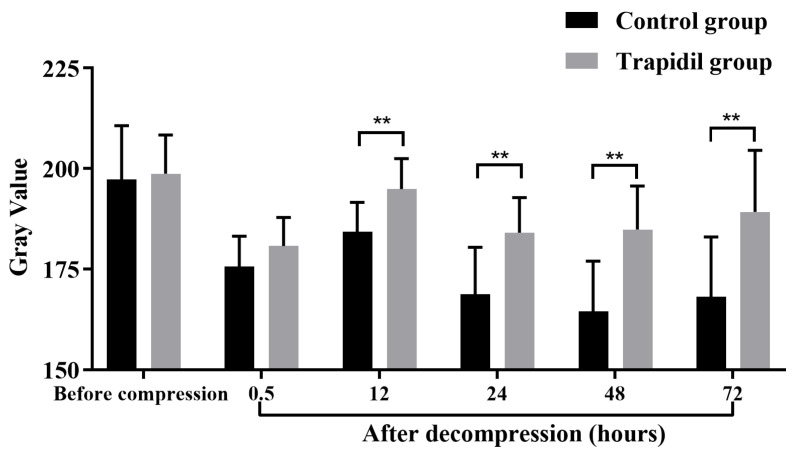
The gray value in control and Trapidil groups. ** *p* < 0.01.

Previous studies have shown that the gray value (GV) can be calculated from photos taken by UV cameras, which can predict the changes in PIs, and the lower the GV of PIs, the higher the severity of PIs [24]. As shown in Figure 6, at 12 h (*p* < 0.01), 24 h (*p* < 0.01), 48 h (*p* < 0.01), and 72 h (*p* < 0.01) after decompression, the GVs in the Trapidil group were significantly higher than those in the control group, and the differences were statistically significant.

#### 2.2.4. Macroscopic Observation and Spectroscopic Measurement of Control and Trapidil Groups after EB Injection

**Figure 7:**
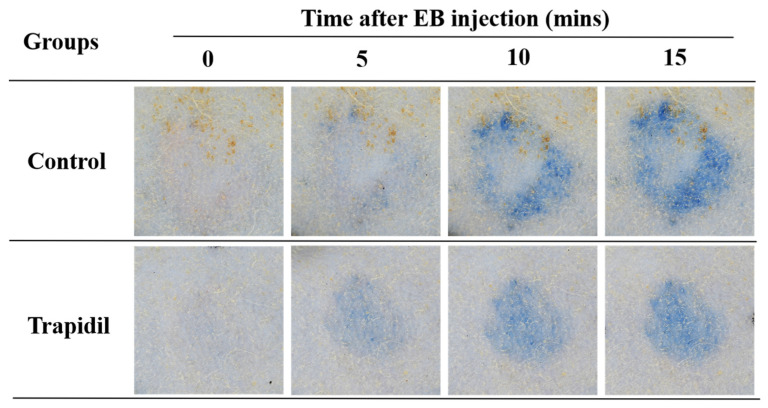
Macroscopic observation of control and Trapidil groups after EB injection. EB: Evans Blue.

**Figure 8:**
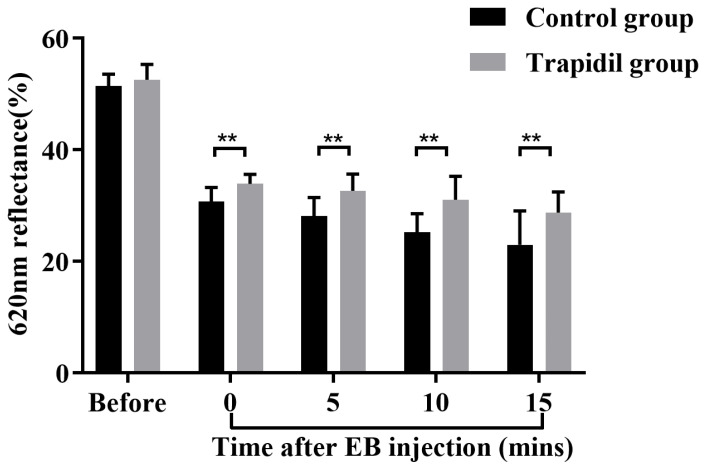
Spectroscopic measurement of control and Trapidil groups after EB injection. EB: Evans Blue. ** *p* < 0.01.

As shown in Figure 7, those in both the control and Trapidil groups gradually darkened in color after EB injection. The control group had a deeper periphery, a shallower interior, and a larger area. The overall color of the Trapidil group was darker than normal skin but lighter than the peripheral part of the control group. Figure 8 shows the spectral measurement of the control and Trapidil groups after EB injection. The reflectivity of both groups showed a decreasing trend, indicating that the colors in both groups became darker, which was consistent with the macroscopic observation results. The reflectivity of the control group was lower than that of the Trapidil group at various time points after EB injection.

#### 2.2.5. The Skin Temperature in Control and Trapidil Groups

**Figure 9:**
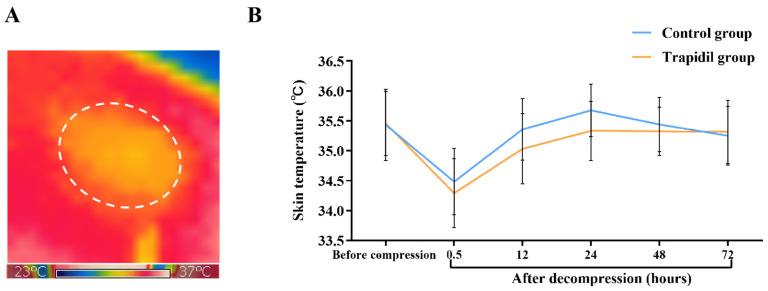
The skin temperature of control and Trapidil groups. (**A**) Evaluation of the skin temperature using infrared-ray thermography non-contact thermometer. (**B**) The difference in skin temperature over time between control and Trapidil groups.

Under normal conditions, the skin temperature of the control group was (35.43 ± 0.56) °C and that of the Trapidil group was (35.45 ± 0.51) °C. From 30 min to 48 h after decompression, the temperature in the control group was higher than that in the Trapidil group. Although none of the differences were statistically significant, at 24 h after decompression, the skin temperature in the control group was (35.68 ± 0.44) °C and that in the Trapidil group was (35.33 ± 0.49) °C, *p* = 0.087 (Figure 9).

#### 2.2.6. Skin Moisture in Control and Trapidil Groups

**Figure 10:**
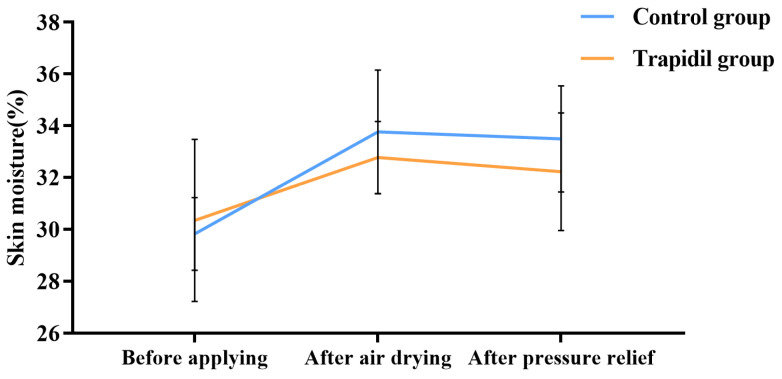
The skin moisture of control and Trapidil groups.

As shown in Figure 10, regarding skin moisture, there was no significant difference between the control and Trapidil groups at all observation points (*p* = 0.60, 0.23, and 0.17, respectively).

## 3. Discussion

The results of this study confirmed that the prophylactic application of Trapidil, which is a water-soluble antiplatelet drug with a low molecular weight, at the compression site can be used against PIs. This is because Trapidil can reduce platelet aggregation, then reduce thrombosis, increase blood supply, and reduce the damage caused by ischemia. Before carrying out the experiments on the topical skin application of Trapidil against PIs, this study proved that the permeability of water-soluble substances in normal skin was very poor, including small-molecular-weight tracers below 500 Da, through experiments on the penetration of water-soluble tracers with different molecular weights into the skin. However, in PI skin, the penetration of a tracer with a molecular weight of 374 Da was significant, and the tracer with a slightly higher molecular weight of 567 Da could also enter the skin, but the tracer with a molecular weight of 961 Da could not easily enter. This also proved that Trapidil, as a water-soluble substance with a small molecular weight, can penetrate into PI skin and effectively exert the effects of antiplatelet aggregation and activation.

The incidence of perioperative pressure injuries remains high. Our recent research found that its pathogenesis is mainly sustained ischemia caused by thrombosis after decompression. The administration of antiplatelet drugs may inhibit this ischemic injury, but, first, it is essential to clarify the transdermal effect of water-soluble drugs. Therefore, in the first part of this study, we used MB (molecular weight is 374 Da), PB (molecular weight is 567 Da), and EB (molecular weight is 961 Da) to compare the permeability of normal skin and PI skin. We found that in normal skin, the permeability of the three tracers was very poor, and almost none of them penetrated the epidermis, even MB, which has a molecular weight below 500 Da. This shows that water-soluble tracers with low molecular weights also cannot easily break through SC; this is consistent with previous research [16]. However, in PI skin, not only could MB with a low molecular weight can enter the dermis in large quantities but PB, which has a molecular weight higher than 500 Da, could also penetrate into the dermis, though the amount was lower than MB. However, EB did not penetrate, which means that in the early stage of PI skin, when no obvious damage is visible to the naked eye, the barrier effect of the epidermis is weakened, but the impact is less significant than that of the adhesive tape test [17]. This is what should be considered when selecting transdermal drug delivery for PI skin. In previous studies, the weakening of the skin barrier function in PI skin can be indirectly observed through methods such as transdermal water loss (TEWL) [25], indicating that the skin’s water retention function is affected before significant changes occur to the naked eye. On this basis, this study directly observed the disruption of the skin barrier function using tracers. This part of the study compared the penetration of water-soluble substances with different molecular weights in normal skin and PI skin for the first time. It was found that water-soluble substances did not easily penetrate normal skin but did penetrate PI skin, especially those with lower molecular weights, which provides a new idea for the percutaneous treatment of PI skin in the future.

**Figure 11:**
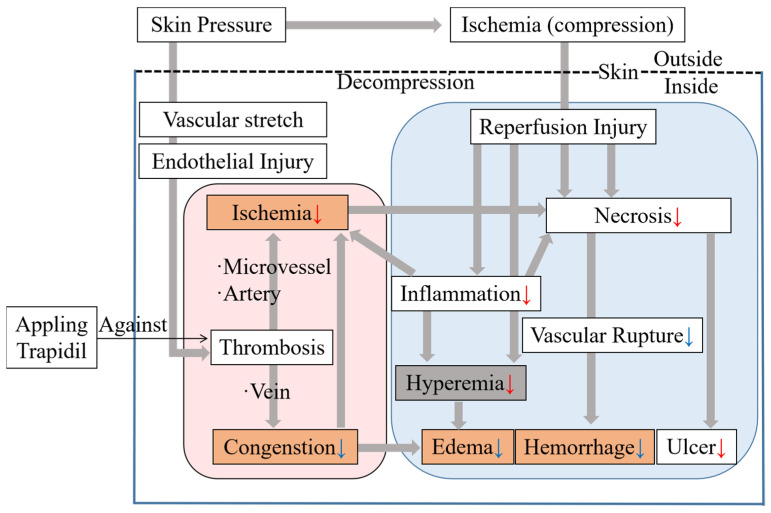
Topical skin application of small-molecule antiplatelet agent against pressure injuries. The mechanism of pressure injuries is from reference [9]. Red arrow (↓) means a reduction compared with the control group in this study. Blue arrow (↓) means a predicted reduction compared with the control group, which needs to be confirmed in future studies.

Trapidil is a water-soluble substance with a molecular weight of 205 Da. In this study, compared with the control group, the Trapidil group displayed a lower incidence and smaller area of ulcers. Previous studies have shown that GVs can be calculated from photos taken by UV cameras, which can predict the changes in PI, and the lower the GV of PI, the higher the severity of PI [24]. The GVs in the Trapidil group were significantly higher than those in the control group. Chen et al. [9] demonstrated severe ischemia in early PI through the intravenous injection of EB in animal experiments. In this study, after the intravenous injection of EB, it was found that the peripheral color of the compressed area in the control group was darker, possibly due to congestion, and the internal color was lighter, possibly due to ischemia. The overall color in the Trapidil group was darker than normal skin but lighter than the peripheral part in the control group. The reflectivity of the Trapidil group was higher than that of the control group at all time points. In addition, the skin temperature in the Trapidil group was lower, indicating that congestion was less significant than that in the control group. These results are mainly due to the fact that Trapidil can reduce platelet aggregation and activation and inhibit thrombosis by increasing prostaglandin synthesis and inhibiting thromboxane synthetase [19]. As shown in Figure 11, the skin underwent tissue deformation after compression, leading to endothelial cell damage by pulling blood vessels, resulting in platelet aggregation and thrombosis. In this study, applying Trapidil reduced platelet aggregation and thrombosis, which can reduce ischemia and tissue damage, thus reducing the inflammatory reaction and necrotic tissue and acting against PI. In this study, water-soluble Trapidil was applied to the skin and then the skin was air-dried. As for skin moisture, there was no significant difference between the control and Trapidil groups at any observation point, indicating that coating and air-drying water-soluble substances in the Trapidil group did not affect skin moisture. Trapidil also has a vasodilator effect. Generally, after vasodilation, the skin temperature will increase. However, in this study, the skin temperature in the Trapidil group did not increase. Therefore, it is preliminarily judged that its vasodilator effect had little effect on the experimental results. However, whether its vasodilator effect also has an effect on PI skin remains to be further explored in future research. In the future, the time and method of administration will be further studied in order to significantly improve the efficacy of Trapidil when applied locally.

As in complementary methods used to predict the progress of PI in previous studies, a UV camera was used to detect the degree of hemorrhage [24], and a spectrophotometer was used to evaluate the skin blood volume [9]. These two methods were also implemented in this study. A UV camera can accurately detect the degree of hemorrhage by observing the changes in reflectivity, as changes in reflectivity are an effective indicator to predict the progress of early PI [24]. The two methods were highly likely to diagnose PI from the perspective of blood volume. From the results, both methods were likely to accurately diagnose early PI, but the UV camera was more convenient and efficient in clinical use.

## 4. Materials and Methods

### 4.1. Animals and Construction of PI Rat Models

#### 4.1.1. Animals

In this experiment, 10-week-old healthy male hairless rats (HWY/Slc, SLC, Inc., Shizuoka, Japan) were used. They were given standard food and water. The experiment was conducted in accordance with recommendations in the Guide for the Care and Use of Laboratory Animals of the National Institutes of Health. The first (experiments on the penetration of water-soluble tracers with different molecular weights into the skin) and second parts of this study (experiments on topical skin application of Trapidil against PIs) were both approved by the ethics committee of Animal Experiments of Nagano College of Nursing, numbered 2022-1 and 2022-2, respectively. 

#### 4.1.2. Construction of PI Rat Models

The construction of PI rat models followed the method proposed by Chen et al. [9]. The hairless rats were anesthetized with an intraperitoneal injection of a mixture of medetomidine hydrochloride (0.15 mg/kg; Domitor, Zenoaq, Fukushima, Japan), midazolam hydrochloride (2.0 mg/kg; Dormicaum; Astellas Pharma, Tokyo, Japan), and butorphanol (2.5 mg/kg; Vetorphale; Meiji Seika Pharma Co., Ltd., Tokyo, Japan). After that, the skin on the back of the rat was pinched up, and the skin was clamped with a 10 mm diameter circular neodymium magnet (Niroku Seisakusho Co., Ltd., Kobe, Japan) with a pressure of 440 mmHg for 3 h and 50 min. To prevent direct contact with the skin, the compression side of the skin with a magnet was attached with gauze. In addition, a commercially available oily magic pen was used to mark the head, tail, and back of the pressure injuries for positioning.

### 4.2. Experiments on Penetration of Water-Soluble Tracers with Different Molecular Weights into Skin

#### 4.2.1. Three Tracers with Different Molecular Weights

In order to determine the penetration of water-soluble substances with different molecular weights into the skin, three tracers with different molecular weights were selected and applied to the skin in this study. Table 2 shows the molecular weight, maximum absorbance, and reflectivity measured using the spectrophotometer of three tracers with different molecular weights.

#### 4.2.2. Grouping and Protocol in First Part of the Study

**Figure 12:**
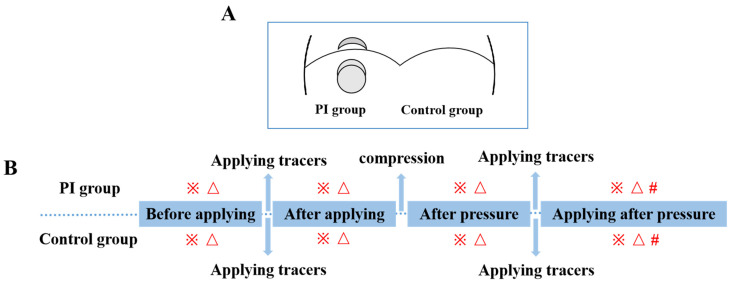
Grouping (**A**) and protocol (**B**) in first part of the study. PI: pressure injury. ※: macroscopic observations. △: determination of reflectivity by spectrophotometer. #: histological observation.

To achieve the maximum penetration effect, the concentrations of the tracers were limited to the maximum dissolution amount. That is, 5 g of EB and PB were each dissolved in 25 mL of distilled water, and 5 g of MB was dissolved in 50 mL of distilled water. In this experiment, a total of 9 rats were used, and each rat was compressed with two magnets on a part of its back, which could produce 2 PIs. Further, 3 rats were smeared with EB solution, 3 rats were smeared with PB solution, and 3 rats were smeared with MB solution. As shown in Figure 12A, the left side of the PI model was called the PI group (*n* = 18, including EB = 6, PB = 6, MB = 6), and the right side without the PI model was called the control group (*n* = 18, including EB = 6, PB = 6, MB = 6). Tracers were applied to the backs of rats on both sides before compression. To prevent drying, PARAFIRM (American National Can) was pasted on the application site, and it infiltrated for 10 min. Then, the PARAFIRM was removed, and the skin was air-dried for 10 min. After this, the PI model was constructed on the left side of each rat’s back. The gauze on the surface of the magnet also adsorbed tracers to maintain adequate penetration of tracers during compression. After decompression, tracers were applied again; then, PARAFIRM was pasted again and infiltrated for 10 min. Finally, the residual liquid was wiped off. Figure 12B is the protocol of this part of the study.

#### 4.2.3. Macroscopic Observations

The skin condition of the rat was photographed using a digital camera (Opti WG-3 PENTAX, Tokyo, Japan) before and after applying tracers and before and after pressure. Macro images of three different tracers applied to normal skin and PI skin were taken. The distance between the camera and the skin was fixed at 20 cm with a fixed device, and the shooting magnification was 2 times. The whole PI skin was photographed.

#### 4.2.4. Determination of Reflectivity Using Spectrophotometer

In order to establish the amount of tracer remaining on the skin, this study used a spectrophotometer (NF555 NIPPON DENSHOKU, Tokyo, Japan) to measure the reflectivity. Because the absorbance of the three tracers is different, we chose different absorbances for different tracers in actual measurements, as shown in Table 2.

#### 4.2.5. Histological Observation

In this study, in order to understand the penetration depth of different tracers, after the euthanasia of rats, the skin tissues were frozen and embedded in OCT compound and cut into 40 μM thin sections in a low-temperature environment and then observed using an unstained optical microscope.

### 4.3. Experiments on Topical Skin Application of Trapidil against PI

#### 4.3.1. Grouping and Protocol in Second Part of the Study

**Figure 13:**
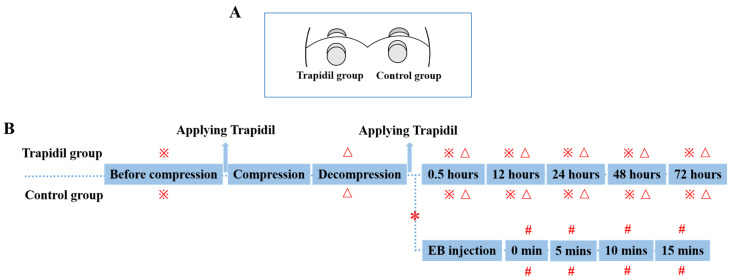
Grouping (**A**) and protocol (**B**) in second part of the study. ※: macroscopic observations, UV observations, measurement of gray value, skin temperature, skin moisture. △: measurement of occurrence rates and areas of ulcers. #: macroscopic observation and spectroscopic measurement. *: Another set of Trapidil and control groups (previous steps were the same).

Ten pieces of Trapidil (MOCHIDA PHARMACEUTICAL Co., Ltd., Osaka, Japan) of 100 mg/piece were crushed, then dissolved in 40 mL of distilled water (maximum dissolved amount) and applied to the left side of the back of rats. To prevent drying, PARAFIRM was pasted on the application site, and it was infiltrated for 10 min. Then, the PARAFIRM was removed, and the skin was air-dried for 10 min. Then, the PI model was built using the methods mentioned above. After relieving the pressure, Trapidil was applied on the same part where Trapidil had just been applied. To prevent drying, PARAFIRM was pasted on the application site, and it was infiltrated for 10 min. Then, the residual liquid was wiped. As shown in Figure 13A, in this experiment, a total of 6 rats were used, and two magnets compressed one area to produce 2 PIs. Therefore, each rat had 4 PIs; the 6 rats had a total of 24 PIs. The left side coated with Trapidil was called the Trapidil group (*n* = 12), and the right side without Trapidil was called the control group (*n* = 12). Figure 13B is the protocol of this part of the study. As for another set of Trapidil (*n* = 8) and control groups (*n* = 8), the previous steps were followed.

#### 4.3.2. Macroscopic Observations

The skin condition visible to the naked eye was recorded using a digital camera (Opti WG-3 PENTAX, Tokyo, Japan). The distance between the camera and the skin was fixed at 20 cm with a fixed device, and the shooting magnification was 2 times. The whole PI skin was photographed.

#### 4.3.3. UV Dermal Camera Photography

It was reported that UV dermal cameras can predict PI [24]. The images of the area and color concentration of PI over time were also captured using a UV camera (DZ-D100, CASIO, Tokyo, Japan) and then measured using image analysis software (ImageJ ver.1.47, National Institutes of Health). Based on previous research [24], the severity of PI can be evaluated using GVs. GVs can be used to indicate the color concentration from 0 to 255. Higher values indicate a darker color on the UV camera, indicating more serious PIs. In skin with PIs, the area where the skin ruptured and formed ulcers was also measured.

#### 4.3.4. Evaluation of Skin Circulation and Perfusion

In order to evaluate the skin circulation and perfusion, half an hour after decompression, EB solution (200 mg/kg) was injected through the femoral vein in this experiment, which is a biomedical marker used to estimate blood volume [26]. The reflectivity was measured at 620 nm with a spectrophotometer (based on EB’s maximum absorbance).

#### 4.3.5. Determination of Skin Surface Temperature

After positioning the PI sites, a full picture of the PI was taken. An FLIR i3 infrared-ray thermography non-contact thermometer (FLIR i3 Thermal Imager, FLIR Systems, Inc., Wilsonville, OR, USA) was used to evaluate the temperature of the skin with PIs.

#### 4.3.6. Skin Moisture Measures

In order to evaluate skin moisture levels, a DM-R2 skin moisture scanner (Panasonic Co., Tokyo, Japan) was used to measure the left and right PI sites at three time points, namely, normal, after drying, and decompression, in the control and Trapidil groups.

### 4.4. Statistical Analysis

The data were analyzed using IBM SPSS Statistics 26.0 software. A Chi-square test was used for counting data. If the measurement data conformed to a normal distribution, they were described with mean ± standard deviation and compared with a *t*-test. If they did not conform to a normal distribution, a nonparametric test was used. *p* < 0.05 indicated that the difference was statistically significant.

## 5. Conclusions

This study found that the topical application of a water-soluble small-molecule antiplatelet agent called Trapidil on the skin can act against PIs. It can relieve ischemia by inhibiting thrombosis, without affecting the overall coagulation function of patients, providing a new idea for the prevention and treatment of PIs in the future. In addition, this study also found that PI skin with an impaired barrier function is more conducive to the penetration of water-soluble substances than normal skin, which also provides a new perspective for the external application treatment of PIs in the future.

## 6. Limitations

This study has some limitations. First, we applied the small-molecule antiplatelet drug to rats, and it showed an anti-PI effect. Whether it is suitable for humans requires further discussion. In addition, further research is needed regarding the drug concentration and frequency of use to maximize the efficacy of the drug.

## Figures and Tables

**Table 1:** Occurrence rates of ulcers in control and Trapidil groups.

After Decompression	Control Group	Trapidil Group
0 min	0%	0%
30 min	0%	0%
12 h	0%	8.33%
24 h	16.67%	16.67%
48 h	83.33%	25.00%
72 h	83.33%	41.67%

**Table 2:** The molecular weight, maximum absorbance, and reflectivity measured by spectrophotometer of three tracers with different molecular weights.

Tracers	Molecular Weight (Da)	Maximum Absorbance (nm)	Reflectivity Measured by Spectrophotometer (nm)
Evans Blue (EB)	961	610	620
Patent Blue (PB)	567	640	640
Methylene Blue (MB)	374	660	660

## Data Availability

The data presented in this study are available on request from the corresponding author.
